# Epidermal Growth Factor Receptor Mutations and Their Prognostic Value with Carcinoembryonic Antigen in Pathological T1 Lung Adenocarcinoma

**DOI:** 10.1155/2018/2942618

**Published:** 2018-04-24

**Authors:** Wang-Yu Zhu, Hai-Feng Li, Ke-Xin Fang, Bing-Jie Zhang, Shi-Quan Zhou, Yong-Kui Zhang, Han-Bo Le, Xiao-Fei Hu

**Affiliations:** ^1^Cellular and Molecular Biology Laboratory, Zhoushan Hospital of Wenzhou Medical University, Zhoushan, Zhejiang, China; ^2^Lung Cancer Research Centre, Zhoushan Hospital of Wenzhou Medical University, Zhoushan, Zhejiang, China; ^3^Department of Cardio-Thoracic Surgery, Zhoushan Hospital of Wenzhou Medical University, Zhoushan, Zhejiang, China; ^4^Department of Respiratory Medicine, Zhoushan Hospital of Wenzhou Medical University, Zhoushan, Zhejiang, China

## Abstract

**Aims:**

The prognostic value of epidermal growth factor receptor (*EGFR*) mutations in the context of serum carcinoembryonic antigen levels remains controversial in T1 lung adenocarcinoma.

**Methods:**

Clinical and pathological characteristics, preoperational carcinoembryonic antigen levels, *EGFR* mutations, and disease-free and overall survival were analysed retrospectively in 573 pathological T1 patients in East China.

**Results:**

*EGFR* mutations were detected in 220 of 573 patients (38.4%). Patients with serum carcinoembryonic antigen levels ≥ 2.12 ng/mL had worse disease-free (*P* < 0.001) and overall survival (*P* < 0.001) than had others, although survival was comparable between patients with and without *EGFR* mutations. However, patients with exon 21 mutations in *EGFR* had significantly better overall survival than had patients with exon 19 mutations (*P* = 0.016), although disease-free survival was comparable (*P* = 0.424). Among patients with serum carcinoembryonic antigen levels ≥ 2.12 ng/mL, disease-free (*P* = 0.019) and overall survival (*P* < 0.001) was also better than that in those with exon 21 mutations. Finally, the exon 19 deletion was found to be an independent predictor of unfavourable overall survival (*P* = 0.037).

**Conclusions:**

*EGFR* mutations were associated with preoperational serum carcinoembryonic antigen levels ≥ 2.12 ng/mL. In patients with levels above this threshold, those with the exon 19 deletion have less favourable prognosis than have those with the exon 21 mutation.

## 1. Introduction

Lung adenocarcinoma is the most prevalent subtype of non-small cell lung cancer, and the 5-year overall survival remains poor [1–3]. Lung adenocarcinoma may arise from an accumulation of genetic mutations, of which those in epidermal growth factor receptor (*EGFR*) are some of the most important and are associated with tumour progression, proliferation, and survival [4]. *EGFR* mutations are the most common genetic lesions in adenocarcinoma but are very rare in squamous cell carcinoma [5]. However, the median progression-free survival in squamous cell carcinoma patients treated with *EGFR* tyrosine kinase inhibitors is worse than that in adenocarcinoma patients with *EGFR* mutations [6]. Thus, *EGFR* mutations may be predictive of the therapeutic response to such inhibitors [7]. Similarly, non-small cell lung cancer patients with mutated *EGFR* also have higher median disease-free survival and improved overall survival [8–10]. However, the predictive value of *EGFR* mutations in patients with pathological T1 lung adenocarcinoma is still unclear. Strikingly, deletion of *EGFR* exon 19 and a point mutation in exon 21 account for up to 90% of *EGFR* mutations in the clinic and correspond to two distinct tumour subtypes with different clinical characteristics and response to *EGFR* tyrosine kinase inhibitors [11, 12]. Hence, the prognostic role of *EGFR* mutations in T1 lung adenocarcinoma is probably well-defined.

In contrast, carcinoembryonic antigen has been used as a biomarker of prognosis and therapeutic efficacy in non-small cell lung cancer. Notably, Cai [13] reported that carcinoembryonic antigen levels gradually increase with the rate of *EGFR* mutations. Moreover, carcinoembryonic antigen levels were reported to be independently prognostic in lung adenocarcinoma patients without *EGFR* mutations [14]. Indeed, we also found that lung adenocarcinoma patients with carcinoembryonic antigen levels above 2.12 ng/mL have a poor prognosis [15]. Nevertheless, other surveys indicated that carcinoembryonic antigen levels are normal in most patients with early-stage lung cancer. Hence, the purpose of this study was to investigate *EGFR* mutations in the context of carcinoembryonic antigen levels and to assess the prognostic value of such mutations in patients with pathological T1 lung adenocarcinoma.

## 2. Methods

### 2.1. Patients

Patients who underwent surgical resection for pathological T1 adenocarcinoma of the lung (*N* = 573) were enrolled retrospectively at Zhoushan Hospital, Zhejiang, China, from July 2011 through March 2016. Histological subtypes were assigned by two pathologists, in accordance with World Health Organization classification and new criteria from the International Association for the Study of Lung Cancer, American Thoracic Society, and European Respiratory Society [16]. The staging of all patients with lung cancer was redefined according to the proposed 8th edition of lung cancer classification [17–19]. The maximum diameter of the resected lesion was measured by a pathologist. Patients were also genotyped for *EGFR* mutations. Clinicopathological features including age, sex, comorbidities, smoking history, lymphatic vessel invasion, vascular vessel invasion, pleural invasion, tumour maximum diameter, tumour stage, tumour histologic subtype, and preoperative serum carcinoembryonic antigen levels were analysed systematically. Patients with resected tumours greater than 3 cm in maximum diameter were excluded. Patients with incomplete records and follow-up data were also excluded, along with patients who died within 30 days after surgery. Patients were monitored over time, using computed tomography (CT) to assess recurrence. Overall survival was calculated as the period from surgical resection to end of follow-up, which was considered to be the time of death, or at the final follow-up of surviving patients. Disease-free survival was calculated as the period between surgery and initial detection of recurrence and metastasis. The study was approved by the Ethical Review Committee of the Zhoushan Municipal Government, and written informed consent was obtained from subjects or their families.

### 2.2. Genomic DNA Extraction and *EGFR* Genotyping

Resected tumours were fixed with 10% formalin, embedded in paraffin, and sectioned at 10 *μ*m. Genomic DNA was extracted from five sections using a QIAamp DNA FFPE Tissue Kit (QIAGEN, Hilden, Germany). DNA concentration and purity were assessed on a Quawell Q3000 spectrophotometer (Quawell Technology, Sunnyvale, CA, USA). *EGFR* was genotyped on a 7500 Real-Time PCR System (ABI, Foster City, CA, USA) using an amplification refractory mutation system (Yuanqi Diagnostics, Shanghai, China), following the manufacturer's instructions.

### 2.3. Statistical Analysis

Data were analysed in GraphPad Prism 5.0 (GraphPad Software Inc., San Diego, CA) and SPSS 17.0 (SPSS Institute, Chicago, IL, USA). Pearson's chi-squared test or Fisher's exact test (*t* < 1 or *n* < 40) was performed to compare differences between categorical groups. The Gaussian distribution was examined according to the Kolmogorov-Smirnov test. Then, the Mann–Whitney *U* test [data shown as the median (*P*_25_, *P*_75_)] or unpaired *t*-test (data shown as the mean ± SD) was used to analyse the difference in CEA level between *EGFR* mutated and wild-type patients. Disease-free and overall survival was evaluated by Kaplan-Meier curves. Multivariate analysis was performed using Cox's proportional hazards regression model for all prognostic factors with univariate *P* < 0.05. All statistical tests were two-sided, with *P* < 0.05 considered significant.

## 3. Results

### 3.1. Relationship between *EGFR* Mutations and Clinicopathological Features

**Figure 1:**
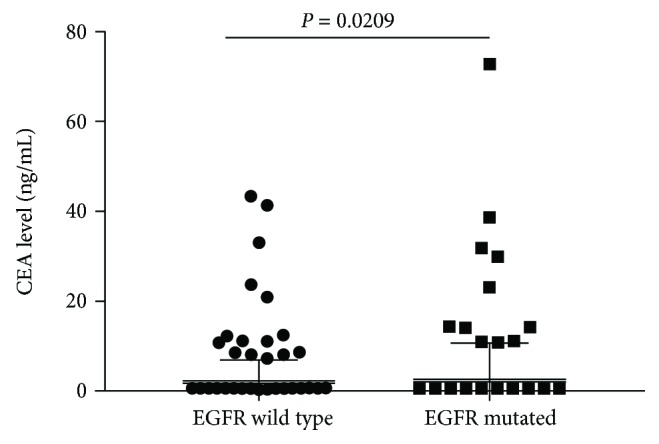
Box and whisker plot of preoperational CEA levels between *EGFR* mutated (*n* = 220) and wild-type (*n* = 353) lung carcinoma patients [1.83 (1.22, 2.91) versus 1.61 (1.05, 2.54), *P* = 0.0209].

Clinical and pathological characteristics are summarized in Table 1 for 573 patients in eastern Chinese islands who had lung adenocarcinoma with a maximum diameter of 3.0 cm or less. Of these patients, 220 were found postsurgery to harbour *EGFR* mutations (38.4%), consisting of G719X in exon 18 (*n* = 6, 1.0%), exon 19 deletion (*n* = 64, 11.2%), exon 20 insertion (*n* = 1, 0.2%), L858R and/or L861Q in exon 21 (*n* = 145, 25.3%), and combined mutations in exons 18 and 19 (*n* = 1, 0.2%), exons 20 and 21 (*n* = 1, 0.2%), and exons 19 and 21 (*n* = 2, 0.3%). The CEA level was higher in *EGFR* mutated patients than in the wild-type patients [1.83 (1.22, 2.91) versus 1.61 (1.05, 2.54), *P* = 0.0209, Figure 1]. Mutations were more likely to occur in patients with carcinoembryonic antigen ≥ 2.12 ng/mL (*P* = 0.030), a threshold identified in our previous survey [15]. Mutations were also associated with tumour size pT1b and pT1c (*P* < 0.001), pleural invasion (*P* = 0.016), histology (*P* < 0.001), lymphatic metastasis (*P* = 0.026), and stage (*P* < 0.001). All other clinical features were comparable between patients with and without *EGFR* mutations (Table 1).

Among patients with carcinoembryonic antigen levels < 2.12 ng/mL (Table 2), *EGFR* mutations were associated with age (*P* < 0.001), tumour size (*P* < 0.001), histology (*P* < 0.001), and stage (*P* < 0.001). In contrast, *EGFR* mutations in patients with carcinoembryonic antigen levels ≥ 2.12 ng/mL were associated with age (*P* = 0.002), nonsmokers (*P* = 0.038), tumour size (*P* = 0.010), histology (*P* < 0.001), and stage (*P* < 0.001, Table 2).

The clinical features of patients with an exon 19 deletion or an exon 21 point mutation are listed in Table 3. Exon 21 point mutations were more common in patients with the lepidic predominant invasive adenocarcinoma (IAC) subtype (*P* = 0.022). However, the frequency of mutations was comparable among other IAC subtypes and was not associated with other clinical and pathological features (Table 3).

### 3.2. Disease-Free and Overall Survival for Pathological T1 Lung Adenocarcinoma

**Figure 2:**
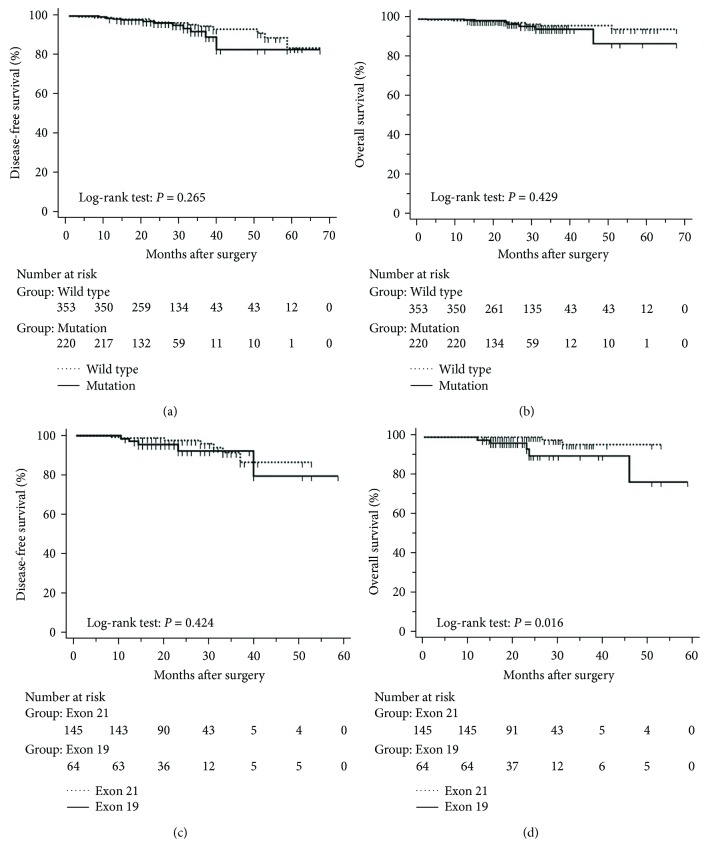
Kaplan-Meier curves after surgery in 573 lung adenocarcinoma patients with tumours with a maximum diameter of 3 cm or less. (a) Disease-free and (b) overall survival stratified by presence and absence of *EGFR* mutations. (c) Disease-free and (d) overall survival stratified by *EGFR* mutations in exon 19 and exon 21.

**Figure 3:**
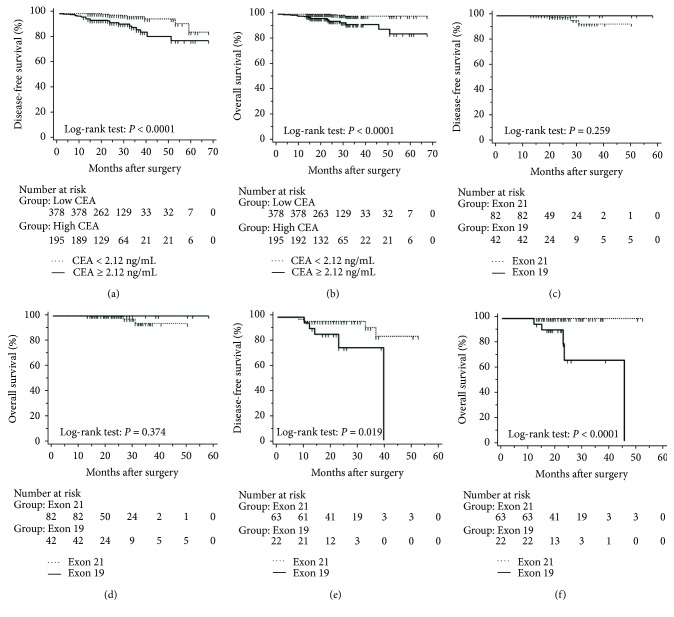
Kaplan-Meier curves after surgery in lung adenocarcinoma patients with tumours with a maximum diameter of 3 cm or less. (a) Disease-free and (b) overall survival stratified by preoperational serum carcinoembryonic antigen levels with a cut-off of 2.12 ng/mL. (c) Disease-free and (d) overall survival in patients with carcinoembryonic antigen levels below 2.12 ng/mL and stratified by mutations in exon 19 and 21. (e) Disease-free and (f) overall survival in patients with carcinoembryonic antigen levels above 2.12 ng/mL and stratified by mutations in exon 19 and 21.

The mean follow-up time was 27.7 months, with a median of 25 months and range of 2.5–68 months. Disease-free and overall survival was comparable between patients with and without *EGFR* mutations. However, patients with exon 21 mutations had significantly better overall survival than had patients with exon 19 mutations (*P* = 0.016), although disease-free survival was comparable (*P* = 0.424, Figure 2). In addition, patients with serum carcinoembryonic antigen levels ≥ 2.12 IU/mL exhibited worse disease-free (*P* < 0.0001) and overall survival (*P* < 0.0001) than did others (Figures 3(a) and 3(b)). Among patients with carcinoembryonic antigen levels below the threshold, disease-free (*P* = 0.259) and overall survival (*P* = 0.374) was comparable between those with exon 21 mutations and exon 19 deletions (Figures 3(c) and 3(d)). Among patients with carcinoembryonic antigen levels above the threshold, disease-free (*P* = 0.019) and overall survival (*P* < 0.0001) was better in those with exon 21 mutations than in those with exon 19 deletions (Figures 3(e) and 3(f)).

### 3.3. Univariate and Multivariate Analyses for Pathological T1 Lung Adenocarcinoma

Univariate and multivariate Cox regression analysis results for disease-free and overall survival are summarized in Table 4. In the multivariate analysis, unfavourable disease-free survival was associated with preoperational carcinoembryonic antigen levels above 2.12 ng/mL (*P* = 0.022), IAC pathology (*P* = 0.046), confirmed lymphatic metastasis (*P* < 0.001), and advanced pathological stage (*P* = 0.033). The exon 19 deletion was an independent predictor of reduced overall survival (*P* = 0.037).

Among patients with carcinoembryonic antigen levels above the threshold (Table 5), exon 19 deletion (*P* = 0.031), large tumour size (*P* = 0.001), IAC pathology (*P* = 0.012), confirmed lymphatic metastasis (*P* = 0.001), and advanced pathological stage (*P* = 0.029) were found by univariate analysis to be unfavourable for disease-free survival. In contrast, large tumour size (*P* = 0.009) and lymphatic metastasis (*P* = 0.003) were predictive of worse overall survival. In the multivariate analysis, none of the clinical features were significantly associated with disease-free survival, although lymphatic metastasis was an independent predictor of reduced overall survival (*P* = 0.039).

### 3.4. Disease-Free and Overall Survival Excluding Stage 0 Patients

**Figure 4:**
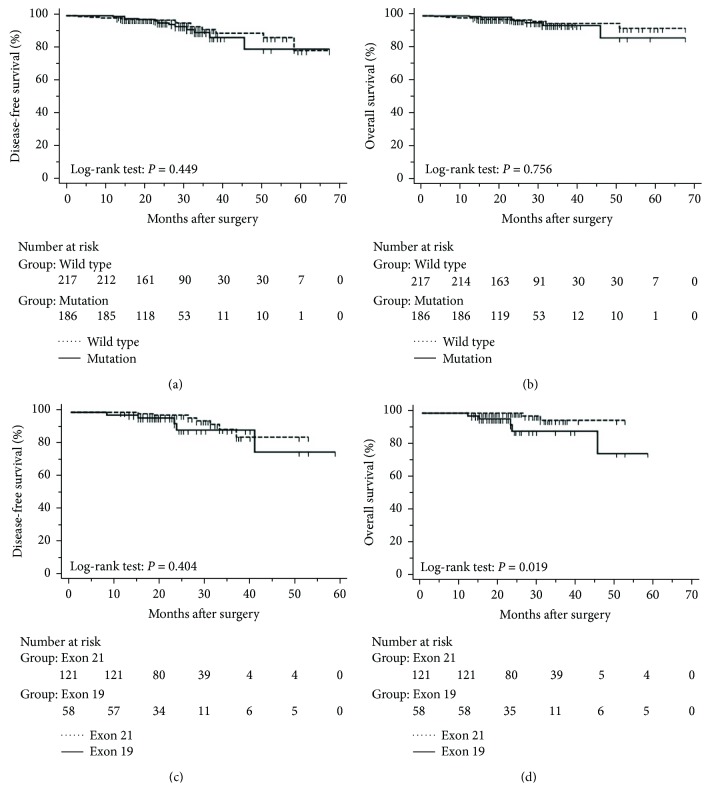
Kaplan-Meier curves after surgery in 403 lung adenocarcinoma patients with tumours with a maximum diameter of 3 cm or less, excluding those at stage 0. (a) Disease-free and (b) overall survival stratified by presence and absence of *EGFR* mutations. (c) Disease-free and (d) overall survival stratified by *EGFR* mutations in exon 19 and exon 21.

**Figure 5:**
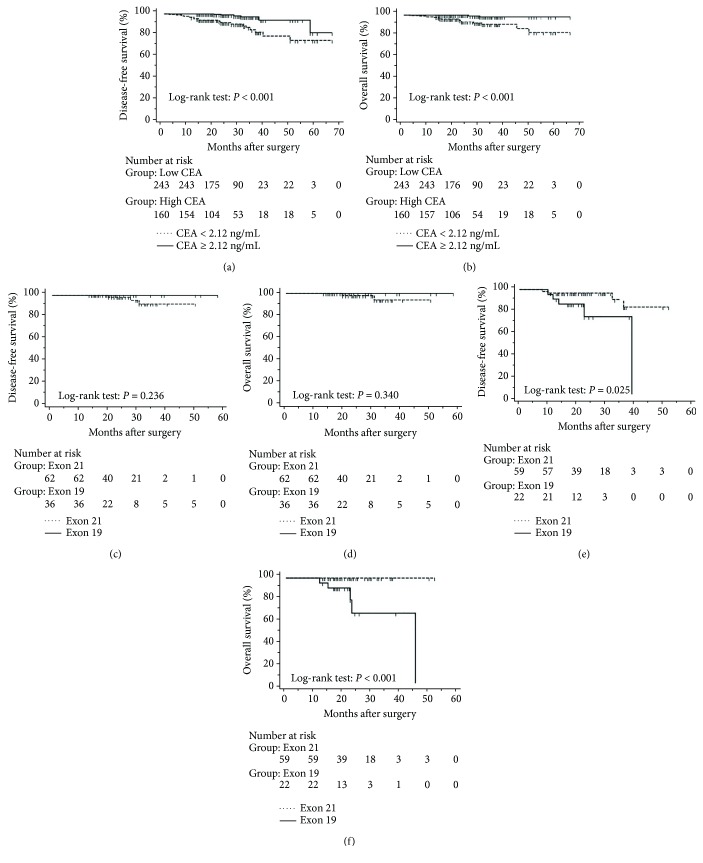
Kaplan-Meier curves after surgery in lung adenocarcinoma patients with tumours with a maximum diameter of 3 cm or less, excluding those at stage 0. (a) Disease-free and (b) overall survival stratified by preoperational serum carcinoembryonic antigen levels with a cut-off of 2.12 ng/mL. (c) Disease-free and (d) overall survival in patients with carcinoembryonic antigen levels below 2.12 ng/mL and stratified by mutations in exon 19 and 21. (e) Disease-free and (f) overall survival in patients with carcinoembryonic antigen levels above 2.12 ng/mL and stratified by mutations in exon 19 and 21.

The progression of lung cancer is typically divided into five stages (0–IV); however, the stage 0 lung cancer patients had better prognosis with 100% disease-free survival and overall survival, and we thus excluded stage 0 patients to analyse the data again [17–19]. The mean follow-up time was 28.0 months, with a median of 25.5 months and range of 2.5–68 months. As shown in Figures 4 and 5, the results were similar to those for the whole cohort, and patients with exon 21 mutations had significantly extended overall survival than had patients with exon 19 mutations (*P* = 0.019, Figure 4). Additionally, patients with a higher level of serum carcinoembryonic antigen levels showed worse disease-free (*P* < 0.001) and overall survival (*P* < 0.001) than did others, and those with exon 21 mutations had better disease-free (*P* = 0.025) and overall survival (*P* < 0.001) than had those with exon 19 deletions (Figure 5).

### 3.5. Univariate and Multivariate Analyses for Pathological T1 Lung Adenocarcinoma

Univariate and multivariate Cox regression analysis results for disease-free and overall survival of patients excluding those in stage 0 are summarized in Table 6. In the multivariate analysis, unfavourable disease-free survival was correlated with preoperational carcinoembryonic antigen levels above 2.12 ng/mL (*P* = 0.031), confirmed lymphatic metastasis (*P* < 0.001), and advanced pathological stage (*P* = 0.012). The exon 19 deletion was an independent predictor of reduced overall survival (*P* = 0.036).

## 4. Discussion

We surveyed the prognostic value of *EGFR* mutations in a cohort of 573 patients from East China who underwent surgical resection of pathological T1 lung carcinoma. In particular, we analysed *EGFR* mutations in patients with preoperational carcinoembryonic antigen levels ≥ 2.12 ng/mL, a key prognostic threshold identified in our previous study [15]. The data suggest that although disease-free and overall survival was comparable between patients with or without *EGFR* mutations, patients with exon 21 mutations had extended overall survival in comparison with patients with exon 19 deletion. Extended disease-free and overall survival was also observed in patients with exon 21 mutations who had carcinoembryonic antigen levels above the threshold, but not for patients with the same mutations who had carcinoembryonic antigen levels below the threshold. Accordingly, exon 19 deletion was an independent predictor of reduced overall survival.

*EGFR* mutations have been reported to be more frequent in Chinese and other Asian populations than in Western populations. Indeed, we detected *EGFR* mutations in 38.4% of our cohort, which was in line with previous surveys of stage I lung adenocarcinoma [20], but slightly lower than the rate in another report [21]. We attribute this difference to the inclusion in our cohort of a number of patients who had tumours of the adenocarcinoma in situ (AIS) subtype (29.7%), in which the frequency of *EGFR* mutations was reported to be 27.3% or 23.8% [22, 23]. In contrast, *EGFR* mutations in our cohort were most prevalent in patients with acinar forms (62.5%), and then in patients with lepidic (56.6%), papillary (54.3%), minimally invasive adenocarcinoma (MIA) (34.1%), solid (22.2%), and AIS (20.0%) forms, in line with other studies [23, 24]. In addition, clinical characteristics were comparable between patients with exon 19 and exon 21 mutations, except that lepidic subtypes were more common in the latter than in the former. Conversely, Japanese and Chinese surveys demonstrated that exon 21 mutations were more common than exon 19 mutations in lepidic tumours [11, 25].

On the basis of our previous study, we stratified the patients by serum carcinoembryonic antigen levels [15]. The new data indicate that serum carcinoembryonic antigen levels ≥ 2.12 ng/mL are associated with *EGFR* mutations, as previously observed in lung cancer [13, 26, 27]. Strikingly, these mutations were more frequent in nonsmokers with serum carcinoembryonic antigen levels ≥ 2.12 ng/mL, even though the prevalence was comparable between smokers and nonsmokers in the entire study population. Notably, we observed that the prevalence was lower at stage 0 than at stages I-III, in contrast to previous surveys [13, 24]. Moreover, we report for the first time that *EGFR* mutations are less frequent in patients with tumour size pT1a than in patients with tumour size pT1b and pT1c. We note, however, that our cohort included a greater proportion of lung adenocarcinoma at stage 0 and tumour size pT1a than had previous cohorts covering stages II to IV. Hence, further prospective surveys are necessary to confirm this result.

Previous surveys of the prognostic value of preoperational serum carcinoembryonic antigen levels have been contradictory, although we note that such surveys use different thresholds [13, 26]. Previously, we determined that levels higher than 2.12 ng/mL were associated with the prognosis of non-small cell lung cancer, and we now report that patients with levels above this threshold had worse disease-free and overall survival than had patients with levels below this threshold. Indeed, carcinoembryonic antigen levels above 2.12 ng/mL were an independent predictor of unfavourable prognosis. Similarly, Yang et al. [25] found that levels above 5 ng/mL were an independent predictor of recurrence-free and overall survival in stage I lung adenocarcinoma. In contrast, in lung adenocarcinoma patients treated with *EGFR* tyrosine kinase inhibitors and platinum-based doublet chemotherapy, carcinoembryonic antigen levels > 5 ng/mL were associated with unfavourable prognosis in patients without *EGFR* mutations, but not in patients with *EGFR* mutations [14, 28]. Strikingly, we found that overall survival, but not disease-free survival, was poorer for patients with exon 21 mutations than for patients with exon 19 mutations. This result contradicts findings in patients with advanced unresectable lung adenocarcinoma who were treated with *EGFR* tyrosine kinase inhibitors but is consistent with a survey by Nishii et al. [29]. In patients with carcinoembryonic antigen levels ≥ 2.12 ng/mL, overall and disease-free survival was also better in patients with exon 21 mutations than in those with exon 19 mutations. However, this relationship was not observed in patients with serum carcinoembryonic antigen levels below 2.12 ng/mL, presumably because the antigen is antiapoptotic. In addition, activation of downstream molecules by *EGFR* mutants may promote antiapoptotic activity, or mutated *EGFR* may elicit abundant expression of the antigen [26, 30]. We also analysed the prognosis of patients excluding those at stage 0; however, the results were similar to those obtained for the overall cohort, which confirms the prognostic role of carcinoembryonic antigen and *EGFR*.

Ultimately, we found that carcinoembryonic antigen levels ≥ 2.12 ng/mL, IAC subtype, lymphatic metastasis, and advanced pathological stage were predictors of worse progression-free survival. Similarly, exon 19 mutations in *EGFR* were an independent predictor of reduced overall survival, especially in patients with carcinoembryonic antigen levels above 2.12 ng/mL. The mechanism underlying the association of carcinoembryonic antigen with *EGFR* mutations remains unclear, and molecular studies are needed to investigate the difference in proliferation and survival between tumours with exon 19 and exon 21 mutations.

Our survey is limited by its retrospective nature, inclusion of several stage 0 patients, small sample size, and patient recruitment in a single institution in Eastern China, which may have resulted in selection bias. Nevertheless, the data imply that in pathological T1 lung adenocarcinoma, *EGFR* mutations are associated with preoperational serum carcinoembryonic levels ≥ 2.12 ng/mL. The data also imply that patients with exon 19 deletions in *EGFR* have less favourable prognosis than had those with exon 21 mutations after curative resection of the lung, especially in patients with carcinoembryonic antigen levels above ≥2.12 ng/mL. Thus, carcinoembryonic antigen levels and *EGFR* genotype should be considered together to assess prognosis in pathological T1 lung adenocarcinoma.

## Figures and Tables

**Table 1:** Clinical and pathologic features of patients with lung adenocarcinoma with a maximum diameter of 3.0 cm or less and stratified by presence and absence of *EGFR* mutations. Where appropriate, data are *n* (%).

Characteristics	All (*n* = 573)	*EGFR* mutated (*n* = 220)	*EGFR* wild type (*n* = 353)	*P*
Age, mean y (range)	57.0 ± 10.0 (26–84)	60.5 ± 10.0 (28–84)	55.0 ± 10.5 (26–77)	<0.001^∗^^a^
Sex				
Male	175 (30.5)	69 (39.4)	106 (60.6)	0.780^b^
Female	398 (69.5)	151 (37.9)	247 (62.1)	
Smoking				
Nonsmoker	468 (81.7)	184 (39.3)	284 (60.7)	0.375^b^
Current/former	105 (18.3)	36 (34.3)	69 (65.7)	
CEA				
<2.12 IU/mL	378 (66.0)	133 (35.2)	245 (64.8)	0.030^∗^^b^
≥2.12 IU/mL	195 (34.0)	87 (44.6)	108 (55.4)	
Type of surgery				
Lobectomy	392 (68.4)	156 (39.8)	236 (60.2)	0.356^b^
Limited	181 (31.6)	64 (35.4)	117 (64.6)	
Tumour size				
pT1a	305 (53.2)	76 (24.9)	229 (75.1)	<0.001^∗^^b^
pT1b	197 (34.4)	103 (52.3)	94 (47.7)	
pT1c	71 (12.4)	41 (57.7)	30 (42.3)	
Histology				
AIS	170 (29.7)	34 (20.0)	136 (80.0)	<0.001^∗^^b^
MIA	167 (29.1)	57 (34.1)	110 (65.9)	
IAC	236 (41.2)	129 (54.7)	107 (45.3)	
IAC subtype (*n* = 236)				
Lepidic	53 (22.5)	30 (56.6)	23 (43.4)	0.041^∗^^c^
Acinar	65 (27.5)	40 (62.5)	25 (37.5)	
Papillary	105 (44.5)	57 (54.3)	48 (45.7)	
Solid	9 (3.8)	2 (22.2)	7 (77.8)	
Mucinous variant	4 (1.7)	0	4 (100.0)	
Pleural invasion				
No	528 (92.1)	195 (36.9)	333 (63.1)	0.016^∗^^b^
Yes	45 (7.9)	25 (55.6)	20 (44.4)	
Lymph nodes				
pN0	546 (95.3)	204 (37.4)	342 (62.6)	0.026^∗^^b^
pN1 or 2	27 (4.7)	16 (59.3)	11 (40.7)	
Pathological stage				
0	170 (29.7)	34 (20.0)	136 (80.0)	<0.001^∗^^c^
IA	339 (59.2)	149 (44.0)	190 (56.0)	
IB	37 (6.4)	21 (56.8)	16 (43.2)	
IIA, IIB, IIIA	27 (4.7)	16 (59.3)	11 (40.7)	

CEA: carcinoembryonic antigen; AIS: adenocarcinoma in situ; MIA: minimally invasive adenocarcinoma; IAC: invasive adenocarcinoma. ^∗^*P* < 0.05 by ^a^*t*-test, ^b^Pearson's chi-squared test, and ^c^Fisher's exact test.

**Table 2:** Clinical and pathological features of T1 lung adenocarcinoma patients stratified by carcinoembryonic antigen (CEA) levels. Where appropriate, data are *n* (%).

Characteristics	CEA < 2.12 IU/mL (*n* = 378)		*P*	CEA ≥ 2.12 IU/mL (*n* = 195)		*P*
	*EGFR* mutated	*EGFR* wild type		*EGFR* mutated	*EGFR* wild type	
Age, mean y (range)	58.4 ± 10.2 (26–83)	52.9 ± 10.5 (26–83)	<0.001^∗^^a^	63.7 ± 8.3 (38–84)	59.8 ± 8.6 (38–84)	0.002^∗^^a^
Sex						
Male	37 (16.3)	190 (83.7)	0.260^b^	55 (49.1)	57 (50.9)	0.143^b^
Female	96 (63.6)	55 (36.4)		32 (38.6)	51 (61.4)	
Smoking						
Nonsmoker	115 (35.0)	214 (65.0)	0.873^b^	69 (49.6)	70 (50.4)	0.038^b^
Current/former	18 (36.7)	31 (63.3)		18 (32.1)	38 (67.9)	
Type of surgery						
Lobectomy	89 (36.3)	156 (63.7)	0.574^b^	67 (45.6)	80 (54.4)	0.738^b^
Limited	44 (33.1)	89 (66.9)		20 (41.7)	28 (58.3)	
Tumour size						
pT1a	58 (23.9)	185 (76.1)	<0.001^∗^^b^	18 (29.0)	44 (80.0)	0.010^∗^^b^
pT1b	60 (53.6)	52 (46.4)		43 (50.6)	42 (49.4)	
pT1c	15 (65.2)	8 (34.8)		26 (54.2)	22 (45.8)	
Histology						
AIS	29 (21.5)	106 (78.5)	<0.001^∗^^b^	5 (14.3)	30 (85.7)	<0.001^∗^^b^
MIA	35 (28.2)	89 (71.8)		22 (51.2)	21 (48.8)	
IAC	69 (58.0)	50 (42.0)		60 (51.3)	57 (48.7)	
IAC subtype (*n* = 236)						
Lepidic	20 (60.6)	13 (39.4)	0.344^c^	10 (50.0)	10 (50.0)	0.123^c^
Acinar	19 (41.3)	27 (58.7)		21 (56.8)	16 (43.2)	
Papillary	30 (76.9)	9 (23.1)		27 (56.3)	21 (43.7)	
Solid				2 (22.2)	7 (77.8)	
Mucinous variant	0	1 (100.0)		0	3 (100.0)	
Pleural invasion						
No	124 (34.3)	238 (65.7)	0.105^b^	71 (42.8)	95 (57.2)	0.231^b^
Yes	9 (56.3)	7 (43.7)		16 (55.2)	13 (44.8)	
Lymph nodes						
pN0	130 (34.8)	244 (65.2)	0.127^c^	74 (43.0)	98 (57.0)	0.267^b^
pN1 or 2	3 (75.0)	1 (25.0)		13 (56.5)	10 (43.5)	
Pathological stage						
0	29 (21.5)	106 (78.5)	<0.001^∗^^c^	5 (14.3)	30 (85.7)	0.001^∗^^c^
IA	93 (41.5)	131 (58.5)		56 (48.7)	59 (51.3)	
IB	8 (53.3)	7 (46.7)		13 (59.1)	9 (40.9)	
IIA, IIB, IIIA	3 (75.0)	1 (25.0)		13 (56.5)	10 (43.5)	

^∗^ *P* < 0.05 by ^a^*t*-test, ^b^Pearson's chi-squared test, and ^c^Fisher's exact test.

**Table 3:** Clinical and pathological features of T1 lung adenocarcinoma patients with *EGFR* exon 19 deletion or exon 21 point mutations. Where appropriate, data are *n* (%).

Characteristics	Exon 19 (*n* = 64)	Exon 21 (*n* = 145)	*P*
Age, mean y (range)	59.7 ± 10.7 (30–80)	61.2 ± 9.4 (28–84)	0.324^a^
Sex			
Male	24 (35.8)	43 (64.2)	0.263^b^
Female	40 (28.2)	102 (71.8)	
Smoking			
Nonsmoker	50 (28.6)	125 (71.4)	0.158^b^
Current/former	14 (41.2)	20 (58.8)	
CEA			
<2.12 IU/mL	42 (33.9)	82 (66.1)	0.218^b^
≥2.12 IU/mL	22 (25.9)	63 (74.1)	
Type of surgery			
Lobectomy	48 (31.6)	104 (68.4)	0.737^b^
Limited	16 (28.1)	41 (71.9)	
Tumour size			
pT1a	16 (24.2)	50 (75.8)	0.231^b^
pT1b	32 (31.1)	71 (68.9)	
pT1c	16 (40.0)	24 (60.0)	
Histology			
AIS	6 (20.0)	24 (80.0)	0.267^b^
MIA	15 (27.8)	39 (72.2)	
IAC	43 (34.4)	82 (65.6)	
IAC subtype (*n* = 125)			
Lepidic	3 (10.7)	25 (89.3)	0.022^∗^^c^
Acinar	16 (40.0)	24 (60.0)	
Papillary	23 (41.8)	32 (58.2)	
Solid	1 (50.0)	1 (50.0)	
Pleural invasion			
No	54 (29.3)	130 (70.7)	0.278^b^
Yes	10 (40.0)	15 (60.0)	
Lymph nodes			
pN0	59 (30.6)	134 (69.4)	0.955^b^
pN1 or 2	5 (31.3)	11 (68.7)	
Pathological stage			
0	6 (20.7)	24 (79.3)	0.216^b^
IA	43 (30.1)	99 (69.9)	
IB	10 (47.6)	11 (52.4)	
IIA, IIB, IIIA	5 (31.3)	11 (68.7)	

CEA: carcinoembryonic antigen; AIS: adenocarcinoma in situ; MIA: minimally invasive adenocarcinoma; IAC: invasive adenocarcinoma. ^∗^*P* < 0.05 by ^a^*t*-test, ^b^Pearson's chi-squared test, and ^c^Fisher's exact test.

**Table 4:** Univariate and multivariate analysis results of disease-free and overall survival in patients with T1 lung adenocarcinoma.

Factor	Univariate analysis			Multivariate analysis		
	HR	95% CI	*P*	HR	95% CI	*P*
*Disease-free survival*						
Sex (female versus male)	2.303	1.111–4.775	0.025^∗^	1.268	0.583–2.758	0.550
CEA (<2.12 versus ≥2.12)	4.999	2.211–11.302	<0.001^∗^	2.877	1.166–7.097	0.022^∗^
Tumour size (pT1a versus pT1b versus pT1c)	2.709	1.664–4.411	<0.001^∗^	1.454	0.786–2.687	0.233
Histology (AIS and MIA versus IAC)	6.300	2.400–16.540	<0.001^∗^	3.204	1.023–10.040	0.046^∗^
Lymphatic metastasis (absent versus present)	8.901	3.934–20.139	<0.001^∗^	11.201	2.658–47.209	0.001^∗^
Pathological stage (stage 0 versus I, II, and IIIA)	2.128	1.430–3.168	<0.001^∗^	0.361	0.163–0.800	0.012^∗^
*Overall survival*						
Sex (female versus male)	3.472	1.320–9.130	0.012^∗^	2.070	0.308–13.899	0.454
Smoking (non versus current/former)	3.235	1.231–8.506	0.017^∗^	1.276	0.129–12.663	0.835
CEA (<2.12 versus ≥2.12)	9.200	2.641–32.049	0.001^∗^	2.626	0.352–19.583	0.346
*EGFR* mutation (exon 21 versus exon 19)	6.170	1.162–32.768	0.033^∗^	7.153	1.124–45.516	0.037^∗^
Tumour size (pT1a versus pT1b versus pT1c)	4.056	2.046–8.039	<0.001^∗^	5.793	0.934–35.944	0.059
Histology (AIS and MIA versus IAC)	21.184	2.187–124.759	0.003^∗^	36416.346	0.000–3.671E220	0.967
Lymphatic metastasis (absent versus present)	9.309	3.275–26.457	<0.001^∗^	14.820	0.172–1276.425	0.236
Pathological stage (stage 0 versus I, II, and IIIA)	2.201	1.319–3.673	0.003^∗^	0.448	0.045–4.468	0.494

HR: hazard ratio; CI: confidence interval; CEA: carcinoembryonic antigen; AIS: adenocarcinoma in situ; MIA: minimally invasive adenocarcinoma; IAC: invasive adenocarcinoma. ^∗^*P* < 0.05.

**Table 5:** Univariate and multivariate analysis results of disease-free and overall survival in patients with carcinoembryonic antigen (CEA) ≥ 2.12 IU/mL.

Factor	Univariate analysis			Multivariate analysis		
	HR	95% CI	*P*	HR	95% CI	*P*
*Disease-free survival*						
*EGFR* mutation (Ex21 versus Ex19)	4.281	1.138–16.105	0.031^∗^	2.705	0.657–11.136	0.168
Tumour size (pT1a versus pT1b versus pT1c)	2.910	1.506–5.623	0.001^∗^	2.813	0.551–14.358	0.214
Histology (AIS and MIA versus IAC)	13.031	1.748–97.143	0.012^∗^	76752.907	0.000–6.609E256	0.970
Lymphatic metastasis (absent versus present)	4.811	1.926–12.018	0.001^∗^	9.748	0.262–362.601	0.217
Pathological stage (stage 0 versus I, II, and IIIA)	1.664	1.054–2.627	0.029^∗^	0.772	0.099–6.005	0.804
*Overall survival*						
Tumour size (pT1a versus pT1b versus pT1c)	2.966	1.318–6.674	0.009^∗^	3.204	0.989–10.382	0.052
Lymphatic metastasis (absent versus present)	5.216	1.725–15.7737	0.003^∗^	2.444	1.044–5.720	0.039^∗^

HR: hazard ratio; CI: confidence interval; AIS: adenocarcinoma in situ; MIA: minimally invasive adenocarcinoma; IAC: invasive adenocarcinoma. ^∗^*P* < 0.05.

**Table 6:** Univariate and multivariate analysis results of disease-free and overall survival in patients with T1 lung adenocarcinoma, excluding those at stage 0.

Factor	Univariate analysis			Multivariate analysis		
	HR	95% CI	*P*	HR	95% CI	*P*
*Disease-free survival*						
Sex (female versus male)	2.320	1.072–5.023	0.033^∗^	1.377	0.601–3.157	0.450
CEA (<2.12 versus ≥2.12)	5.203	2.086–12.977	<0.001^∗^	3.016	1.104–8.241	0.031^∗^
Tumour size (pT1a versus pT1b versus pT1c)	2.235	1.464–3.412	<0.001^∗^	1.580	0.800–3.119	0.188
Histology (MIA versus IAC)	7.971	1.881–33.772	0.005^∗^	1.913	0.224–16.317	0.553
Lymphatic metastasis (absent versus present)	7.183	3.117–16.553	<0.001^∗^	19.612	3.888–98.923	<0.001^∗^
Pathological stage (stage I versus II, and IIIA)	2.054	1.263–3.340	0.004^∗^	0.342	0.147–0.793	0.012^∗^
*Overall survival*						
Sex (female versus male)	3.332	1.208–9.188	0.020^∗^	2.090	0.312–14.004	0.447
Smoking (non versus current/former)	2.817	1.047–7.578	0.040^∗^	1.263	0.127–12.561	0.842
CEA (<2.12 versus ≥2.12)	11.244	2.552–49.537	0.001^∗^	2.643	0.352–19.842	0.345
*EGFR* mutation (exon 21 versus exon 19)	5.974	1.124–31.746	0.036^∗^	7.221	1.133–46.003	0.036^∗^
Tumour size (pT1a versus pT1b versus pT1c)	3.351	1.698–6.613	<0.001^∗^	6.019	1.036–34.958	0.046^∗^
Lymphatic metastasis (absent versus present)	7.147	2.479–20.606	<0.001^∗^	14.645	0.170–1258.671	0.238
Pathological stage (stage I versus II, and IIIA)	1.898	1.011–3.564	0.046^∗^	0.449	0.045–4.503	0.496

HR: hazard ratio; CI: confidence interval; CEA: carcinoembryonic antigen; AIS: adenocarcinoma in situ; MIA: minimally invasive adenocarcinoma; IAC: invasive adenocarcinoma. ^∗^*P* < 0.05.
